# Heavy Metal Accumulation, Tissue Injury, Oxidative Stress, and Inflammation in Dromedary Camels Living near Petroleum Industry Sites in Saudi Arabia

**DOI:** 10.3390/ani12060707

**Published:** 2022-03-11

**Authors:** Jamaan S. Ajarem, Ahmad K. Hegazy, Gamal A. Allam, Ahmed A. Allam, Saleh N. Maodaa, Ayman M. Mahmoud

**Affiliations:** 1 Zoology Department, College of Science, King Saud University, Riyadh 11451, Saudi Arabia; allam1081981@yahoo.com (A.A.A.); maodaa_28@yahoo.com (S.N.M.); 2 Department of Botany & Microbiology, College of Science, King Saud University, Riyadh 11451, Saudi Arabia; akhegazy@yahoo.com; 3 Department of Botany and Microbiology, Faculty of Science, Cairo University, Giza 12613, Egypt; 4 Immunology Section, Department of Microbiology, College of Medicine, Taif University, Taif 21974, Saudi Arabia; gm_allam@yahoo.com; 5 Zoology Department, Faculty of Science, Beni-Suef University, Beni-Suef 62514, Egypt; 6 Department of Life Sciences, Faculty of Science and Engineering, Manchester Metropolitan University, Manchester M1 5GD, UK

**Keywords:** oil industry, lead, cadmium, nickel, vanadium, ROS, inflammatory cytokines, apoptosis

## Abstract

**Simple Summary:**

The petroleum industry is a major source of energy and economic development but can pollute the environment and negatively impact animal and human health. Heavy metals are environmental pollutants and can reach the human body in direct and indirect ways. We investigated the levels of heavy metals in the soil and in the milk, blood, muscle, liver, and kidney of Arabian camels living near a petroleum industry site in the eastern region of Saudi Arabia. Our results revealed increased lead levels, cadmium, nickel, and vanadium in the soil and different tissues of camels. In addition, liver and kidney dysfunction, tissue damage, oxidative stress, inflammation, and apoptosis were reported. These findings may be of public interest and call attention to the assessment of the impact of the petroleum industry on the environment and the health of nearby communities.

**Abstract:**

The petroleum industry can impact the environment and human health. Heavy metals (HMs), including lead (Pb), cadmium (Cd), nickel (Ni), and vanadium (V), are toxic pollutants found in petroleum that can cause several severe diseases. This study investigated the impact of the oil industry on the Arabian camel (*Camelus dromedarius*) in the eastern region of Saudi Arabia, pointing to HMs accumulation, tissue injury, redox imbalance, inflammation, and apoptosis. Soil and camel samples (milk, blood, muscle, liver, and kidney) were collected from a site near an oil industry field and another two sites to analyze HMs. Pb, Cd, Ni, and V were increased in the soil and in the camel’s milk, blood, muscle, liver, and kidney at the polluted site. Serum aminotransferases, urea, and creatinine were elevated, and histopathological alterations were observed in the liver and kidney of camels at the oil industry site. Hepatic and renal lipid peroxidation, pro-inflammatory cytokines, Bax, and caspase-3 were increased, whereas cellular antioxidants and Bcl-2 declined in camels at the oil extraction site. In conclusion, the oil industry caused soil and tissue accumulation of HMs, liver and kidney injury, oxidative stress, and apoptosis in camels living close to the oil extraction site. These findings pinpoint the negative impact of the oil industry on the environment, animal, and human health.

## 1. Introduction

The petroleum industry potentially impacts water, soil, air, and community health [1]. Exposure of the human population can occur during any phase of the oil industry, including extraction, transport, refinery, and crude oil processing [2]. Oil extraction frequently occurs near a human population, and the oil fields have been reported to affect the health and environment of over 600 million people worldwide [2]. The hazardous impact of the oil industry includes several acute and chronic effects evaluated in people living near oil extraction sites. For instance, increased prevalence of neurological and respiratory symptoms, cardiovascular disease, lupus, and rheumatic disease have been identified among a New Mexico community living near an oil drilling site [3]. The altered immunological function has been suggested to increase the higher rates of allergy [4], lupus [3], and liver disease [5] in communities near oil extraction sites. Increased DNA damage and the risk of cancer have been reported by analyzing blood samples of workers in the oil industry in Ecuador [6]. The serious health effects observed in populations near oil extraction sites could be connected to the release of heavy metals (HMs).

Lead (Pb) and cadmium (Cd) are hazardous HM pollutants produced through various human activities, including the oil industry [7]. The two most abundant metals in petroleum are vanadium (V) and nickel (Ni), where their concentrations reach 1600 mg/kg and 340 mg/kg, respectively [8]. The organic matter derived from algae exposed to suboxic conditions represents the main source of V and Ni [9]. During petroleum diagenesis and catagenesis, organic molecules are transformed into geoporphyrins which chelate VO^2+^ and Ni^2+^ to form metalloporphyrins [9]. V is released into the atmosphere through the combustion of oil and coal [10]. Besides the oil industry, V is used in the steel industry to improve corrosion resistance, electronics, and batteries [11]. Given increasing energy demands and technological developments, the exploitation of heavy oils and other petroleum resources has increased, resulting in V mobilization [11]. Schlesinger et al. reported that the levels of V release through the anthropologic activities can exceed that of many other HMs [11]. Although V is not viewed as a serious contaminant, it could be toxic at high concentrations, and liver injury has been demonstrated in rats exposed to V [12]. Accordingly, inhalation of V pentoxide dust resulted in occupational toxicity [13]. In addition, Ni has unique chemical and physical properties and is used in electroplating and in alloys, Ni-Cd batteries, food, and other industrial processes. The widespread use of Ni leads to the emission of high levels into the environment, causing contamination [14]. Human exposure to Ni-polluted environments may provoke various adverse effects, such as lung fibrosis, renal and cardiovascular disorders, respiratory tract cancer, and others [14].

The presence of HMs in the drilling waste discharges poses a risk of contaminating the environment. Accumulation of HMs in the soil adversely affects its properties and could restrict the biodegradation of organic contaminants. In addition, the continuous leaching of HMs can contaminate subsurface water resources and poses a major risk to human health [15]. Proximity to oil wells in Italy was associated with an accumulation of HMs in the liver and kidney of animals [16]. HMs are non-degradable, and their bioaccumulation and biomagnification could trigger a negative impact on the ecosystem and provoke serious health problems [17,18,19]. Some HMs function as micronutrients and are involved in vital metabolic and cellular processes; however, they are toxic beyond their physiological concentrations [20]. The toxic effects of HMs have been demonstrated in numerous studies [21,22,23,24], with neurotoxicity, cancer, hepatotoxicity, reproductive toxicity, cancer, osteoporosis, and other disorders among the effects of chronic exposure to HMs [17,25,26,27,28,29]. Increased production of reactive oxygen species (ROS) and oxidative injury have been implicated in the toxicity of HMs [30,31]. Excessive ROS can damage the cellular macromolecules, including DNA, lipids, and proteins, and activate NF-kB, resulting in the release of pro-inflammatory mediators, cellular dysfunction, and injury [32].

Given the hazardous impact of the oil industry, this study investigated the concentration of some HMs in different tissues of camels (*Camelus dromedarius*) in the eastern region of Saudi Arabia. In addition, we evaluated the histological changes, oxidative stress biomarkers, inflammation, and apoptosis in the liver and kidney of camels.

## 2. Materials and Methods

### 2.1. Collection of Samples

**Figure 1:**
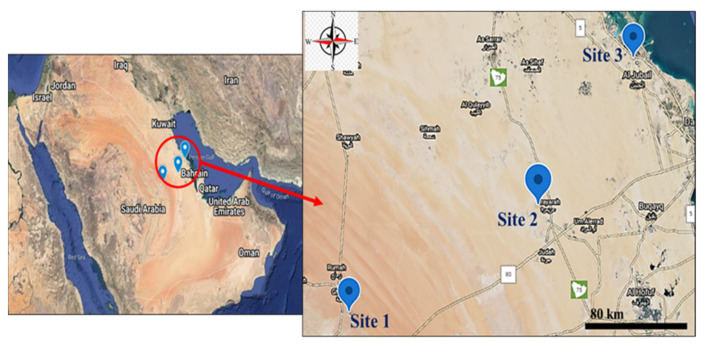
A map showing the sites of the study.

Thirty soil samples were collected from three different regions in the eastern region of KSA to assay HMs contamination. In addition to the soil samples, blood, milk, muscle, liver, and kidney samples were collected from 30 *C. dromedarius* along a distance gradient from the pollution source (petroleum industry site located in the industrial zone of Al-Jubail; Site 3: 27°00′00.0″ N 49°34′57.3″ E), Urayarah (Site 2: 25.9807° N, 48.8498° E) and Rodaht Khoraim (Site 1: 25°22′19.4″ N 47°17′10.9″ E, about 100 km from Riyadh as a reference site) (Figure 1). Site 3 is located 4 km away from the industrial zone in Al-Jubail, and this area contains several sheep pens. Al-Jubail is a city in the Eastern province of Saudi Arabia and home to the Middle East’s largest petrochemical company. The camels (3–4 years) were examined by an experienced veterinarian and were found healthy. The skin at the upper part of the neck was disinfected with iodine alcohol, and blood was collected from the jugular vein with a sterile syringe. The blood was left to coagulate, centrifuged, and serum was separated for measuring aminotransferases, creatinine, and urea, whereas other samples along with milk, muscle, liver, and kidney were processed to determine Pb, Cd, V, and Ni. The muscle, liver, and kidney samples were collected from the camels after scarification in the abattoir for human consumption. Samples from the liver and kidney were fixed in 10% neutral-buffered formalin, and other samples were kept in RNAlater for RNA isolation. Other liver and kidney samples were homogenized (10% *w*/*v*) in cold phosphate-buffered saline (PBS). The homogenate was centrifuged, and the clear supernatant was separated to determine malondialdehyde (MDA), a marker of lipid peroxidation (LPO), and the antioxidants (reduced glutathione (GSH), superoxide dismutase (SOD), and catalase (CAT)).

### 2.2. Determination of Soil and Tissue HMs

Pb, Cd, Ni, and V were determined in the collected soil, milk, blood, and tissue samples using ELAN 9000 ICP-MS (Perkin Elmer Sciex Instruments, Concord, ON, Canada). In this assay, a 200 mg sample was mixed with 2 mL nitric acid in a clean digestion beaker and heated at 140 °C for 40 min. The digest was filtered, and ultrapure water was added to bring the filtrate volume to 10 mL. A blank digest was prepared in the same way. For calibration and quality control, standard references (Aristar grade, VWR International Ltd., Leicestershire, UK) were used. The linear rank of the methodology was assured by analyzing different standards for each element, and all standards were used in duplicate to determine the precision of the analysis. CP-MS detects the elements at a parts-per-trillion level. Ultrapure water was used to prepare blanks and calibration standards, and three replicate determinations were performed for each sample.

### 2.3. Determination of Aminotransferases, Creatininne, and Urea

Levels of ALT, AST, creatinine, and urea were assayed in blood samples collected from camels at the three studied sites with Spinreact (Girona, Spain) reagent kits according to the provided instructions.

### 2.4. Detremination of MDA and Antioxidants

To determine MDA levels in the liver and kidney, a 200 µL sample was mixed with 400 µL thiobarbituric acid (TBA; 0.6%) and 1.2 mL O-phosphoric acid (1%). The mixture was heated for 45 min at 95 °C, 0.8 mL n-butanol was added, followed by vortexing for 1 min and centrifugation for 10 min at 2000 rpm. The upper layer was separated, and the absorbance was measured at 535 nm [33]. GSH content was determined based on its reaction with 5,5′-dithiobis (nitrobenzoic acid) to generate a yellow product. The product absorbance was measured at 412 nm [34]. SOD activity was assayed based on its ability to inhibit pyrogallol autoxidation [35], and CAT activity was determined by continuously monitoring the decomposition of hydrogen peroxide and the decrease in absorbance at 240 nm for 3 min [36]. Protein content was determined using Bradford’s reagent [37].

### 2.5. Measurement of Pro-Inflammatory Cytokines

TNF-α and IL-1β were assayed in the liver and kidney samples using specific ELISA kits (MyBiosource, San Diego, CA, USA) as per the manufacturer’s protocol.

### 2.6. Histolopathological Examination

The fixed liver and kidney samples were processed for paraffin embedding, and 5μm sections were cut using a microtome. The sections were processed for staining with hematoxylin and eosin (H&E) as previously described [38] and examined using a light microscope.

### 2.7. Gene Expression

The liver and kidney samples were crushed into a fine powder with a mortar and a pestle under liquid nitrogen. Total RNA was isolated from the powdered samples using an RNA Purification Kit (Thermo Scientific, Pleasanton, CA, USA) according to the manufacturer’s instructions. The extracted RNA was quantified using a nano-drop, and samples with A260/A280 ≥ 1.8 were selected. One µg RNA was reverse transcribed into cDNA using a high-capacity cDNA reverse transcription kit (Thermo Scientific, Pleasanton, CA, USA). The synthesized cDNA was amplified using Maxima SYBR Green/ROX qPCR master mix (Thermo Scientific, Pleasanton, CA, USA) in 10 µL reaction volume and the primers listed in Table 1, using PPIA as the internal control gene due to its stability and robust CT value [39]. The obtained data were analyzed using the 2^−ΔΔCt^ method [40].

### 2.8. Statistical Analysis

The data were presented as the mean ± standard deviation (SD). The normality of the data was checked with the Shapiro-Wilk test. The statistical comparisons were performed using two-way ANOVA followed by Tukey’s post-hoc test on GraphPad Prism 8. A *p* value < 0.05 was considered statistically significant.

## 3. Results

### 3.1. HMs in Soil Samples

**Figure 2:**
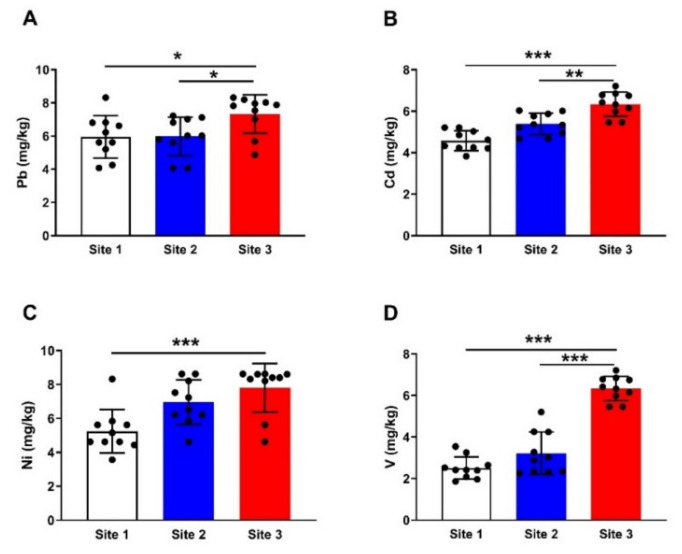
Pb (**A**), Cd (**B**), Ni (**C**), and V (**D**) concentrations in soil samples. Data are mean ± SD (*n* = 10). * *p* < 0.05, ** *p* < 0.01 and *** *p* < 0.001.

Analysis of Pb concentration revealed an increase in soil samples collected from site 3 compared to sites 1 and 2 (*p* < 0.001; Figure 2A). Cd showed a significant increase in soil samples collected from site 3 when compared with samples from site 1 (*p* < 0.001) and site 2 (*p* < 0.01) (Figure 2B). Site 3′s soil showed a significant increase (*p* < 0.001) in Ni (Figure 2C) and V (Figure 2D) concentrations when compared with the reference site (Figure 2).

### 3.2. HMs in Different Tissues of Camels

**Figure 3:**
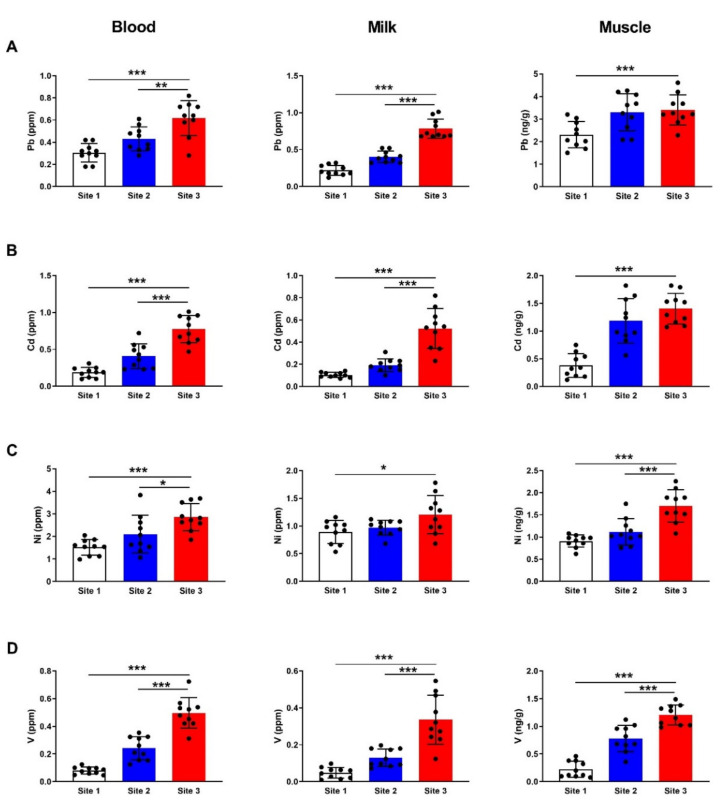
Concentrations of Pb (**A**), Cd (**B**), Ni (**C**), and V (**D**) in blood, milk and muscle of camels at the studied sites. Data are mean ± SD (*n* = 10). * *p* < 0.05, ** *p* < 0.01 and *** *p* < 0.001.

The results revealed a significant increase in Pb in blood and milk samples collected from camels at the polluted site (site 3) when compared with samples from animals at site 1 (*p* < 0.001; *p* < 0.01) and site 2 (*p* < 0.001; *p* < 0.001) as depicted in Figure 3A. Similarly, Pb accumulated significantly in the muscle of camels at site 3 as compared to the reference site (*p* < 0.001; Figure 3A). Cd exhibited a significant increase in blood, milk, and muscle samples (*p* < 0.001) in camels at the polluted site, as represented in Figure 3B. Camels at site 3 showed significant increase in blood (*p* < 0.001), milk (*p* < 0.05) and muscle (*p* < 0.001) Ni levels as compared to samples collected from the reference site (Figure 3C). Analysis of V concentration revealed a significant (*p* < 0.001) increase in the blood, milk, and muscle samples from camels at site 3 (Figure 3D).

**Figure 4:**
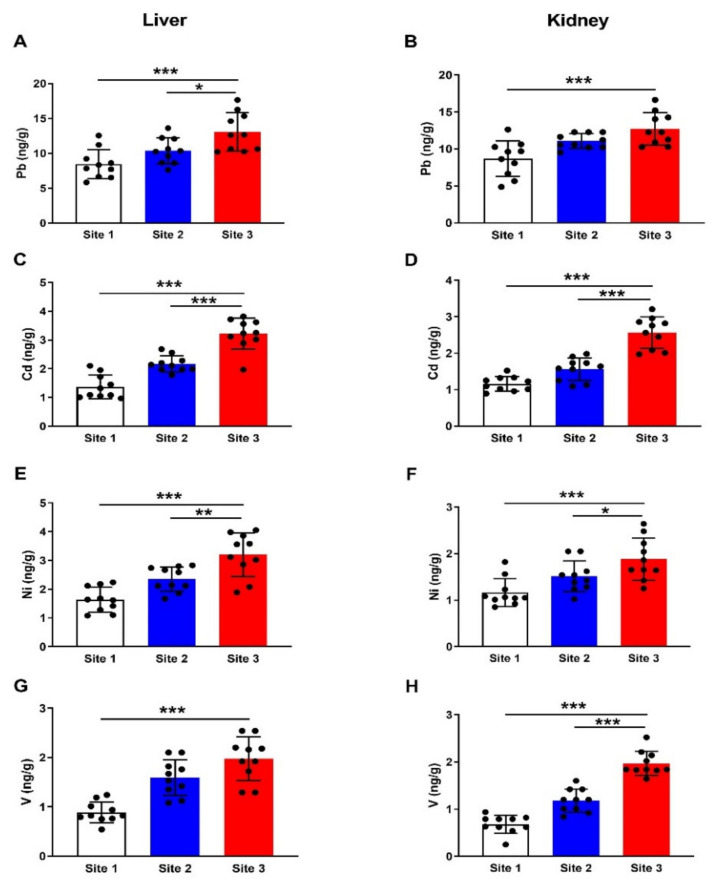
Concentrations of Pb (**A**,**B**), Cd (**C**,**D**), Ni (**E**,**F**), and V (**G**,**H**) in liver and kidney of camels at the studied sites. Data are mean ± SD (*n* = 10). * *p* < 0.05, ** *p* < 0.01 and *** *p* < 0.001.

The concentration of Pb in the liver (Figure 4A) of camels at site 3 showed a significant increase (*p* < 0.001) as compared to the reference site samples. Hepatic Cd (Figure 4B), Ni (Figure 4C), and V (Figure 4D) were significantly increased in site 3 samples as compared to the reference site (*p* < 0.001). Like the liver, all HMs showed significant increases in the kidney samples collected from camels at site 3 compared with the corresponding reference site samples (Figure 4A–D).

### 3.3. Liver and Kidney Function Markers

**Figure 5:**
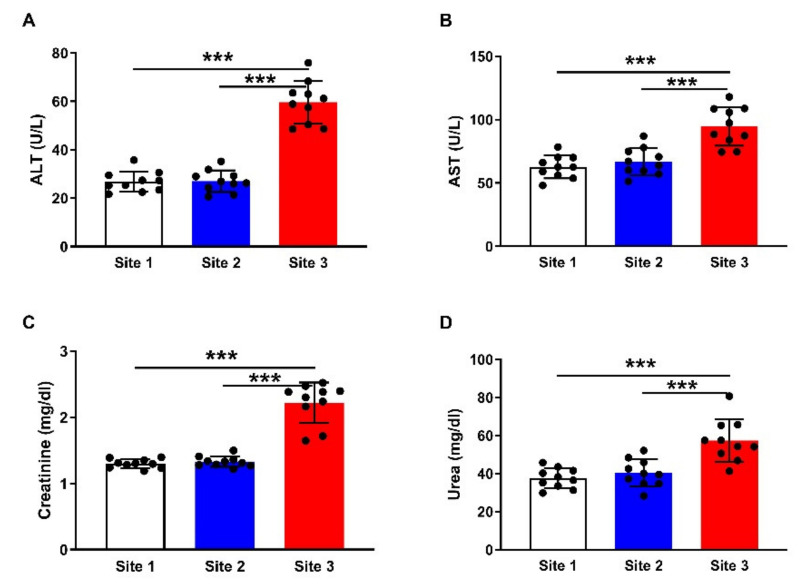
Serum ALT (**A**), AST (**B**), creatinine (**C**), and urea (**D**) of camels at the studied sites. Data are mean ± SD (*n* = 10). *** *p* < 0.001.

To assess the probable negative impact of the accumulated HMs on the liver and kidney function in camels, serum ALT (Figure 5A), AST (Figure 5B), creatinine (Figure 5C), and urea (Figure 5D) were determined. The results showed significant (*p* < 0.001) elevation in the aminotransferases, creatinine, and urea in the serum of camels at site 3 when compared with the other two sites.

### 3.4. Histological Investigation

**Figure 6:**
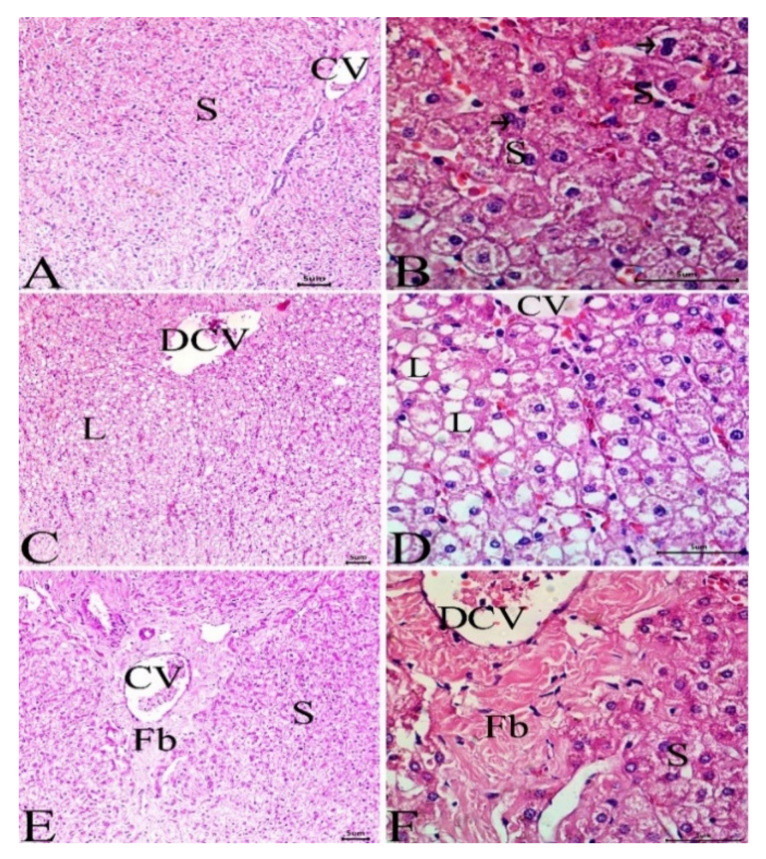
Sections in the liver of camels at site 1 (**A**,**B**) showing normal liver architecture with normal hepatocytes (arrow), sinusoids (S), and central vein (CV), site 2 (**C**,**D**) showing mild dilation of the central vein (DCV) and few lipid droplets (L) and site 3 (**E**,**F**) showing dilated central vein (DCV), lipid droplets and fibrotic changes (Fb). (H&E, Scale bar = 50 μm).

**Figure 7:**
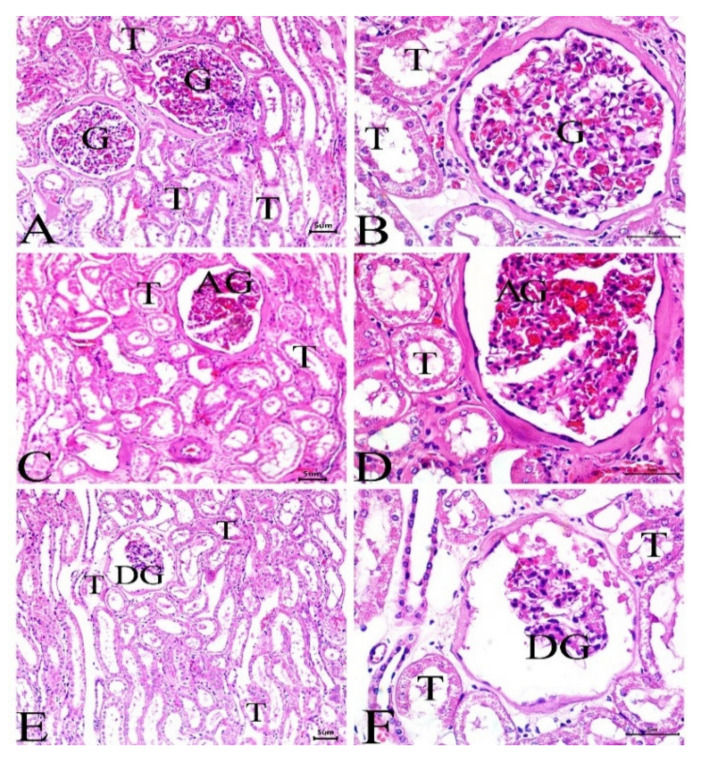
Sections in the kidney of camels at site 1 (**A**,**B**) showing the normal structure of the glomeruli (G) and renal tubules (T), site 2 (**C**,**D**) showing mild glomerular abnormalities (AG), and site 3 (**E**,**F**) showing abnormal and degenerated glomeruli (DG). (H&E, Scale bar = 50 μm).

Liver and kidney samples were collected from camels living at the 3 sites, and sections were prepared, stained, and examined. While animals at site 1 (Figure 6A,B) showed normal liver architecture with normal hepatocytes, sinusoids, and central vein, sections from the liver of animals at site 2 (Figure 6C,D) showed mild dilation of the central vein and few lipid droplets. The liver of camels at site 3 exhibited dilated central vein, lipid droplets, and hepatic tissue fibrosis (Figure 6E,F). The kidney showed abnormal and degenerated glomeruli along with other histopathological manifestations in camels at site 3 (Figure 7E,F), whereas camels at site 2 (Figure 7C,D) revealed mild glomerular abnormalities, whereas the reference site camels showed a normal structure of the glomeruli and renal tubules (Figure 7A,B).

### 3.5. MDA and Antioxidants

**Figure 8:**
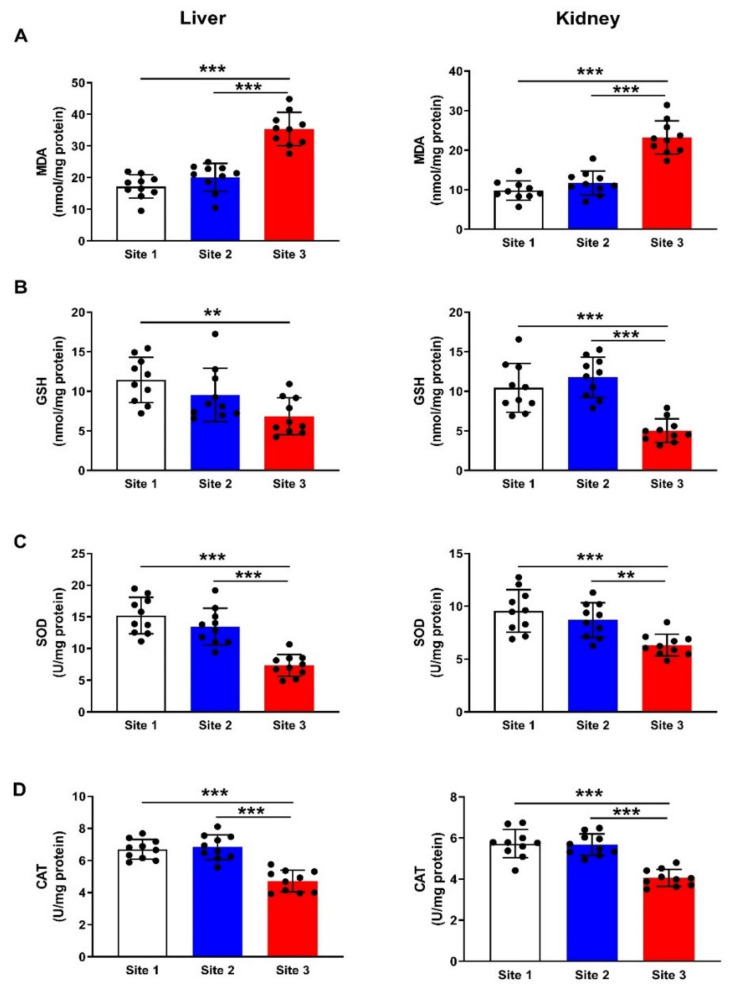
Levels of MDA (**A**) and GSH (**B**), and activities of SOD (**C**) and catalase (**D**) in the liver and kidneys of camels at the studied sites. Data are mean ± SD (*n* = 10). ** *p* < 0.01 and *** *p* < 0.001.

**Figure 9:**
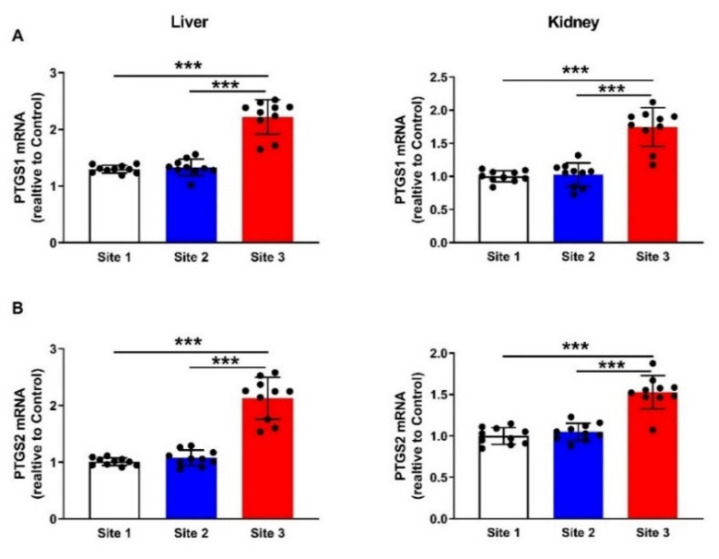
PTGS1 (**A**) and PTGS2 (**B**) mRNA levels in the liver and kidney of camels at the studied sites. Data are mean ± SD (*n* = 10). *** *p* < 0.001.

To evaluate redox imbalance in the liver and kidney of camels at site 3 as compared to the camels at sites 1 and 2, we determined the levels of MDA (Figure 8A) and GSH (Figure 8B) and activities SOD (Figure 8C) and CAT (Figure 8D). MDA was increased in the liver (*p* < 0.001) and kidney (*p* < 0.001) of camels at site 3, whereas GSH, SOD, and CAT were declined. In addition, we determined the mRNA abundance of PTGS1 (Figure 9A), and PTGS2 (Figure 9B), and both were upregulated in the liver and kidney of camels at site 3 when compared with the corresponding camels at sites 1 and 2 (*p* < 0.001).

### 3.6. Inflammatory Cytokines

**Figure 10:**
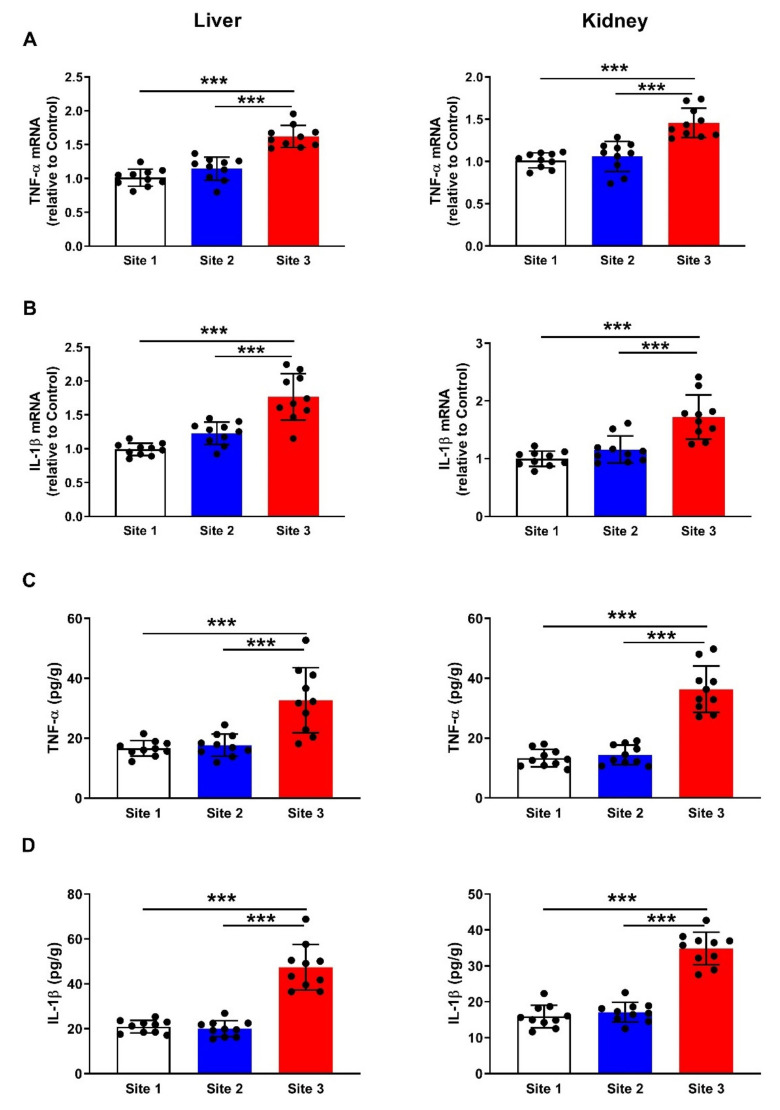
Liver and kidney TNF-α (**A**) and IL-1β (**B**) mRNA abundance, and TNF-α (**C**) and IL-1β (**D**) protein levels of camels at the studied sites. Data are mean ± SD (*n* = 10). *** *p* < 0.001.

mRNA abundance of TNF-α (Figure 10A) and IL-1β (Figure 10B) was upregulated in the liver and kidney of camels at site 3 as compared to the reference site and site 2 (*p* < 0.001). In support of the gene expression data, the protein levels of TNF-α (Figure 10C) and IL-1β (Figure 10D) were elevated significantly in the liver (*p* < 0.001) and kidney (*p* < 0.001) of camels at site 3 as compared to the other sites.

### 3.7. Apoptosis Markers

**Figure 11:**
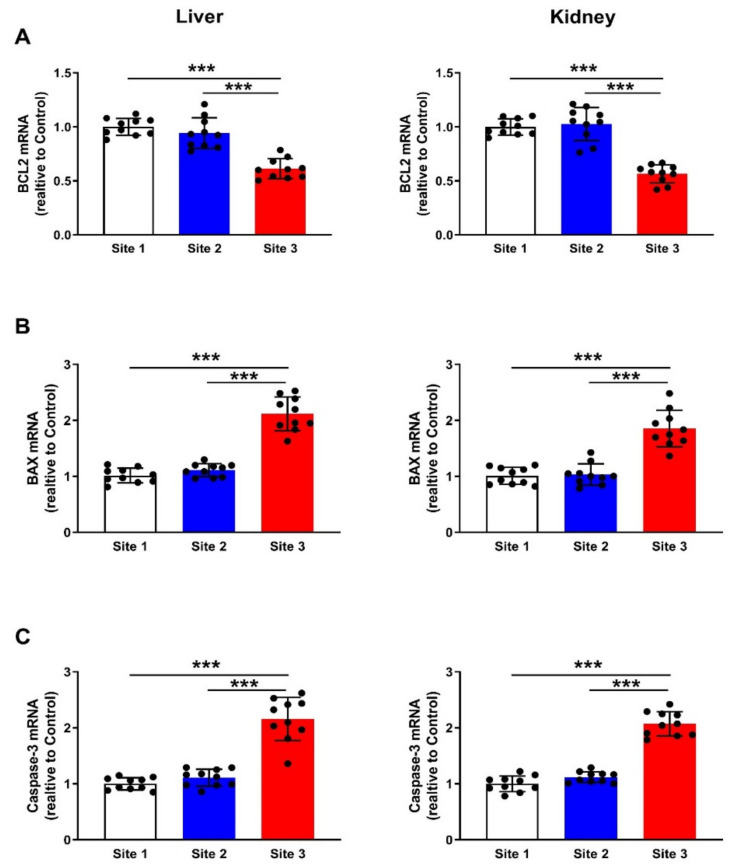
Liver and kidney Bcl-2 (**A**), Bax (**B**), and caspase-3 (**C**) mRNA abundance of camels at the studied sites. Data are mean ± SD (*n* = 10). *** *p* < 0.001.

Apoptosis was evaluated in the liver and kidney of camels through the determination of the mRNA abundance of Bcl-2 (Figure 11A), Bax (Figure 11B), and caspase-3 (Figure 11C). The results revealed remarkable downregulation of Bcl-2 (*p* < 0.001) and significant upregulation of Bax (*p* < 0.001) and caspase-3 (*p* < 0.001) mRNA levels in both liver and kidney of camels at site 3.

## 4. Discussion

Environmental contamination with HMs released from oil extraction, mining, stone quarrying, and other industries occurs commonly in Saudi Arabia and can negatively impact animal and human health [41,42,43]. This study investigated the negative impact of the oil industry in the eastern region of Saudi Arabia on dromedary camels with an emphasis on the concentration of HMs in different tissues and alterations in the liver and kidney. Owing to the role of redox imbalance in mediating the deleterious effects of HMs, oxidative stress markers, antioxidants, inflammatory cytokines, and apoptosis markers were assessed in the liver and kidney of dromedary camels. Analysis of HMs in the soil was carried out to determine the emission and distribution of HMs at the oil extraction site. The results showed a significant increase in the concentration of Pb, Cd, Ni, and V in the soil samples collected from the oil industry site compared with the other sites. Increased soil HMs can negatively affect the soil properties and decrease crop yield. Additionally, the leaching of HMs from the soil to subsurface water resources can reach humans and hence represents a risk of several disorders [15].

Pb, Cd, Ni, and V were increased in the blood, milk, meat, liver, and kidney of camels near the oil extraction site, demonstrating the negative effect of this industry on both camels and human beings. Exposure of the human body to HMs could occur by consuming contaminated water, plants, meat, and dairy products. HMs can easily be transmitted to animals via consuming plants growing in contaminated soil. Here, HMs were increased in the blood, milk, and meat of the camel samples collected from the site of the oil industry. Therefore, the consumption of camel milk and meat could be a route of transmitting these HMs to the body of people living near the oil industry site, particularly children who consume a large amount of milk. In this context, Norouzirad et al. have reported an increase in Pb and Cd levels in cow milk collected from areas near petroleum extraction industries in Southwest Iran [7].

Accumulation of Pb, Cd, Ni, and V in the liver and kidney of camels at the contaminated site was associated with functional and histological alterations. Assessment of the liver and kidney function markers revealed a remarkable elevation in serum ALT, AST, creatinine, and urea, demonstrating liver and kidney injury. In addition, dilated central vein, lipid infiltration, and fibrous deposition were observed in the liver, and abnormal glomeruli and interstitial hemorrhage were observed in the kidneys of camels at the contaminated site. The altered liver and kidney function and the tissue injury could be directly connected to the accumulation of HMs in these organs. Pb and Cd are hazardous HM pollutants produced through various human activities, including the oil industry [7]. Pb is a toxic HM that poses a negative impact on both the environment and human health [44]. The liver and kidneys are the main depositories of Pb within the body [45]. In addition, Cd is a non-degradable HM that can remain in the soil for decades [46,47]. Cd has a high rate of soil-to-plant transfer and can reach the animals and human body through the consumption of plants growing in contaminated soil [48]. Therefore, liver and kidney injury observed in this study is very likely explained by the accumulation of Pb and Cd. In support of our findings, the study of Miedico et al. showed that the proximity to oil wells in southern Italy increased hepatic Pb and renal Cd concentrations in sheep [16]. In another study conducted by Brown, Pb and Cd were increased in different organs of slaughtered animals in oil-producing regions in Colombia [49]. Moreover, rats exposed to V exhibited liver injury [12], and inhalation of V pentoxide dust was associated with occupational toxicity [13]. Renal, cardiovascular, and pulmonary disorders are among the deleterious effects of exposure to Ni-polluted environments [14]. In experimental animals, exposure to Ni induced hepatotoxicity [50] and nephrotoxicity [51].

Oxidative stress is a major player in mediating the toxic effects of HMs. The current investigation revealed a significant increase in hepatic and renal MDA along with declined GSH, SOD, and CAT in camels at the polluted site, demonstrating an oxidative stress status. In addition, PTGS1 and PTGS2, a part of the MDA synthesis pathway, were upregulated in the liver and kidney of camels at the oil extraction site. The negative impact of Pb on the liver and kidney has been demonstrated to occur via oxidative stress in experimental rodent models [52,53,54]. The ionic properties and promotion of ROS production are the main factors contributing to Pb toxicity [55]. Through its ability to replace mono- and bivalent cations, Pb alters metabolism, signaling enzymatic activities and ionic transportation in the cells [56]. Following exposure, Cd reaches the liver bound to albumin, where it provokes ROS generation, GSH depletion, and hepatocyte membrane damage, leading to cell death [31]. In addition, Cd can bind to metallothionine-producing complexes that cause hepatocyte injury and accumulate in and damage the kidney when transported via the circulating blood [57]. Cd can negatively affect the antioxidant system via its ability to bind the thiol group of the antioxidant enzymes [58], and inhibit complex III of the mitochondrial electron transport chain [58]. Kidney injury manifested by glomerular degeneration has been observed in rodents exposed to Cd [59], and hepatotoxicity and several liver diseases are serious effects of exposure to this HM [60]. The toxic effects of Ni are associated with increased ROS, MDA, and NO levels, mitochondrial dysfunction, and swelling in the ovaries of rats [61]. Excessive V has been shown to cause cardiac injury via provoking ROS generation and mitochondrial dysfunction [62]. Very recently, Xiong et al. reported the development of oxidative stress and mitochondrial quality control disorder in the heart of ducks [63]. Our findings added support to the key role of oxidative stress in mediating the toxicity of HMs and introduced new information that petroleum industry-derived HMs causes liver and kidney injury associated with redox imbalance in camels.

Besides oxidative stress, inflammatory response and apoptotic cell death contributed to liver and kidney injury in camels at the petroleum industry site. Here, TNF-α and IL-1β, both mRNA and protein, were remarkably upregulated in the liver and kidney of camels at the polluted site. In addition, Bax and caspase-3 were upregulated, whereas Bcl-2 was downregulated at the oil industry site. These findings show the development of hepatic and renal inflammatory response and apoptosis along with oxidative stress and accumulation of Pb, Cd, Ni, and V. The developed inflammation and apoptosis are a direct consequence of HMs accumulation and increased ROS generation. Excessive ROS can activate NF-κB, a transcription factor that elicits the transcription and release of pro-inflammatory mediators, including TNF-α and IL-1β. ROS and pro-inflammatory cytokines activate Bax and mitochondrial dysfunction, resulting in the release of cytochrome *c* and subsequent activation of caspase-3, which elicits apoptotic cells death [64].

The role of Pb, Cd, V, and Ni in provoking cell death via apoptosis has been well-acknowledged. For instance, Cd-induced apoptosis is associated with decreased mitochondrial membrane potential and Bcl-2/Bax ratio, and activation of caspase-3, in addition to Fas/FasL-mediated mitochondrial apoptotic pathway in neurons [65,66]. The apoptotic effect of Pb is associated with the generation of ROS and activation of inflammatory mediators and caspase-3 [67]. V-induced apoptosis is mediated via the MAPK-Nrf2 pathway in epithelial cells [68] and c-fos in HaCaT cells [69]. Ni elicits the release of mitochondrial cytochrome *c* and promotes the interaction between Fas and FasL, leading to the formation of the death-inducing signaling complex, which contains FADD and procaspase-8 and -10. Within the cells, caspase-8 and -10 cleave and activate caspase-3, -6, and -7, leading to apoptosis [70]. The lack of data on the body weight, age, length measurements, and coat color of the camels is considered a limitation of this study. However, this is an unavoidable limitation when working with non-model species.

## 5. Conclusions

This study introduced new information on the hazardous effect of the petroleum industry in the eastern region of Saudi Arabia on dromedary camels. Pb, Cd, Ni, and V were accumulated in the soil and milk, blood, muscle, liver, and kidney of camels at the oil extraction site, demonstrating the negative environmental impact of this industry. Camels exhibited liver and kidney injuries associated with oxidative stress, upregulated inflammatory and apoptosis markers, and depletion of antioxidants. These findings pointed to the hazardous effects of the petroleum industry on nearby communities, and might be of value for evaluating its negative impact upon them.

## Figures and Tables

**Table 1:** Primers used for qRT-PCR.

Gene	Primers (5′-3′)	GenBank Accession Number	Product Size (bp)
PTGS1	F: CGTGGAGTTCAACCAGCTCTA R: AGGTGTTGAAGAGGAACTGCT	XM_010997991.1	101
PTGS2	F: CTGGTCTGATGATGTACGCC R: ACAACCGCTCATCATCCCAT	XM_010992100.1	99
TNF-a	F: CCAGCTCATGAACCCTCTGG R: GGGTATTGGCAAACCGCTTC	NM_001319880.1	134
IL-1B	F: TGAACCCGCCAGTGAAATGA R: GACGCAGCACTTCATCTGTT	XM_010984994.1	94
BAX	F: CACCAAGGTGCCTGAACTGA R: CGTGGGTGTCCCAAAGTAGG	XM_031459056.1	130
BCL2	F: GTTTGAACTGAGGTACCGGC R: CCCATCCCGGAAGAGTTCAT	XM_010993888.1	115
CASP3	F: GCTTCTTCAGAGGGGACAGT R: TCGGCAGGCCTGAATAATGA	XM_010974562.1	71
PPIA	F: ACCACCAGACCATTCCTTCT R: TATGGAACCCCGAAAACTGC	XM_010987886.1	109

PTGS: prostaglandin-endoperoxide synthase; TNF-a: tumor necrosis factor alpha; IL-1B: interleukin-1beta; CASP3: caspase-3; BCL2: B-cell lymphoma 2; BAX: BCL2 associated X; PPIA: peptidylprolyl isomerase A.

## Data Availability

Data analyzed or generated during this study are included in this manuscript.
